# 

**Published:** 1893-06-10

**Authors:** 


					June 10th, 18?
WITH EXTRA NURSING SUPPLEMENT.
Per Annum, 8s. 6d.t post free.
Monthly Farts, 6d.; post free, 8d.
350. Vol. XIV.] Saturday,
June 10th, 1693.
[Twopence,

				

